# Insights into the Naso-Oropharyngeal Bacterial Composition in Suspected SARS-CoV-2 Cases

**DOI:** 10.3390/pathogens13080615

**Published:** 2024-07-25

**Authors:** Librada A. Atencio, Indira J. Quintero, Alejandro Almanza, Gilberto Eskildsen, Joel Sánchez-Gallego, Mellissa Herrera, Hermógenes Fernández-Marín, José R. Loaiza, Luis C. Mejía

**Affiliations:** 1 Centro de Biodiversidad y Descubrimiento de Drogas, Instituto de Investigaciones Científicas y Servicios de Alta Tecnología (INDICASAT), Clayton, Panama City 0843-01103, Panama; latencio@indicasat.org.pa (L.A.A.); indiravarg@gmail.com (I.J.Q.); aalmanza@indicasat.org.pa (A.A.); hfernandez@indicasat.org.pa (H.F.-M.); 2 Departamento de Microbiología Humana, Facultad de Medicina, Universidad de Panamá, Panama City 0819-07289, Panama; gilberto.eskildsen@up.ac.pa; 3 Department of Marine Earth and Atmospheric Sciences, North Carolina State University, Raleigh, NC 27695, USA; joeljsanchezg@gmail.com; 4 Coiba Scientific Station (COIBA AIP), Gustavo Lara Street, Bld. 145B, City of Knowledge, Clayton, Panama City 0843-01853, Panama; 5 Hospital Luis “Chicho” Fábrega, MINSA, Santiago 0923, Panama; myherrera@minsa.gob.pa; 6 Sistema Nacional de Investigación (SNI), Secretaría Nacional de Ciencia, Tecnología, e Innovación (SENACYT), Panama City 0816-02852, Panama; 7 Smithsonian Tropical Research Institute, Panama City 0843-03092, Panama; 8 Departamento de Genética y Biología Molecular, Universidad de Panamá, Estafeta Universitaria Apartado 3366, Zona 4, Panama City 0819-07289, Panama

**Keywords:** COVID-19, SARS-CoV-2, naso-oropharyngeal microbiome, *Corynebacterium*

## Abstract

Severe acute respiratory syndrome coronavirus 2 (SARS-CoV-2) was the causative agent of the coronavirus disease 2019 (COVID-19) pandemic. While research on COVID-19 has mainly focused on its epidemiology, pathogenesis, and treatment, studies on the naso-oropharyngeal microbiota have emerged in the last few years as an overlooked area of research. Here, we analyzed the bacterial community composition of the naso-oropharynx in 50 suspected SARS-CoV-2 cases (43 detected, 7 not detected) from Veraguas province (Panama) distributed across five age categories. Statistical analysis revealed no significant differences (*p* < 0.05) in bacterial alpha and beta diversities between the groups categorized by SARS-CoV-2 test results, age, or patient status. The genera *Corynebacterium*, *Staphylococcus*, *Prevotella*, *Streptococcus,* and *Tepidiphilus* were the most abundant in both detected and not-detected SARS-CoV-2 group. The linear discriminant analysis effect size (LEfSe) for biomarker exploration indicated that *Veillonella* and *Prevotella* were enriched in detected and hospitalized patients with SARS-CoV-2 relative to non-detected patients, while *Thermoanaerobacterium* and *Haemophilus* were enriched in non-detected patients with SARS-CoV-2. The results also indicated that the genus *Corynebacterium* was found to decrease in patients with detected SARS-CoV-2 relative to those with non-detected SARS-CoV-2. Understanding the naso-oropharyngeal microbiota provides insights into the diversity, composition, and resilience of the microbial community in patients with SARS-CoV-2.

## 1. Introduction

Ever since the COVID-19 outbreak was deemed a global pandemic by the World Health Organization (WHO) on 11 March 2020, severe acute respiratory syndrome coronavirus 2 (SARS-CoV-2) has continued to pose a serious threat to public health across the globe. As of April 2024, over 775 million individuals have been infected globally, resulting in more than seven million fatalities reported by WHO. According to the latest WHO data (https://covid19.who.int/, revised on 22 April 2024), more than 13 billion vaccine doses have been administered thus far. COVID-19 is a systemic disease that can affect multiple organs; however, it primarily targets the respiratory tract. Symptoms caused by SARS-CoV-2 vary among individuals, with respiratory symptoms being the most common, including dyspnea, dry cough, nasal congestion, anosmia, and sore throat [1,2]. Other symptoms such as fever, fatigue, pain, and gastrointestinal illness have also been reported [3]. SARS-CoV-2 is transmitted via the inhalation of laden particles and can enter human cells by binding to angiotensin-converting enzyme 2 receptors. Viral attachment to these receptors initiates the infection process, resulting in inflammation and balance disruption of the upper respiratory tract (URT) microbiota [4,5].

The human microbiota is defined as a set of microorganisms that inhabit and interact with the human body. Microorganisms colonize different sites in the human body where they adapt to niche characteristics. Alterations (dysbiosis) in the human microbiota can increase the susceptibility of the host to infection [6]. The URT is the primary entryway for several respiratory viruses such as SARS-CoV-2, but it also provides different niches for microbial communities. The URT is home to specialized bacterial communities, some of which can safeguard respiratory health by preventing the development and dissemination of pathogens to the lower respiratory tract (LRT) [7]. The naso-oropharyngeal microbiota serves as an essential component of the epithelial barrier and plays an important role in resistance to infection [2,8]. Bacterial taxa, such as *Staphylococcus*, *Corynebacterium*, *Dolosigranulum*, and *Moraxella*, have been reported to be common components of the nasopharyngeal microbiota since early life [9]. Previous studies using 16S rRNA sequencing have demonstrated that alterations in the microbial community during SARS-CoV-2 infection led to a balance disruption in the naso-oropharyngeal microbiota in patients who tested positive for COVID-19 due to the loss of nasal commensal bacteria and an increase in pro-inflammatory bacteria [10]. It has also been reported that the bacterial richness and alpha diversity tend to increase as the disease severity increases, although the alpha diversity decreases in patients with the most severe COVID-19 [11]. Furthermore, these studies suggest that bacterial communities in patients with COVID-19 could play an important role in the clinical diagnosis of the disease severity [12,13].

In this study, we analyzed the bacterial community composition of the naso-oropharynx in suspected SARS-CoV-2 cases from Veraguas province (Panama). More specifically, we addressed the questions of whether subjects with symptoms of COVID-19 classified into one of three groups (SARS-CoV-2 detected (ambulatory and hospitalized), and SARS-CoV-2 not detected) had differences in their naso-oropharyngeal microbial community composition and diversity and whether there were potential bacterial biomarkers of these groups’ condition.

## 2. Materials and Methods

### 2.1. Ethics Statement

This study was approved by the National Bioethics Committee of Research of Panama (EC-CNBI-2020-05-60) to use random de-identified specimens from standard care testing for COVID-19. No prior exclusion criteria were applied.

### 2.2. Sample Collection

Combined naso-oropharyngeal swab (NPS) samples from 50 patients with suspected cases of SARS-CoV-2 infection (i.e., 43 detected and 7 not detected) were collected in the Veraguas Health Region (Province of Veraguas, Republic of Panama) between April and August of 2020, when virus lineages A.2, B.1, and A.1 were SARS-CoV-2 predominant in Panama [14,15,16]. The demographic information for each person sampled was recorded, as well as the result of the virus detection and whether the person was hospitalized. This last feature was used as a proxy for the severity (patient status) of the SARS-CoV-2 infection, divided into three groups: detected ambulatory, detected hospitalized, and not detected (Table S1). Suspected cases of SARS-CoV-2 were classified clinically according to symptoms of respiratory pathology: nasal congestion, 38 °C fever, and dry cough. If suspected patients were positive for SARS-CoV-2, they were classified as detected; if the test result was negative, they were classified as not detected. The SARS-CoV-2 patient status included the following: detected ambulatory (patient with detected SARS-CoV-2 and who did not require hospitalization), detected hospitalized (patient with detected SARS-CoV-2 and who required hospitalization), and not detected (patient who returned a negative SARS-CoV-2 test).

### 2.3. SARS-CoV-2 Detection

The detection of the virus was performed using the GeneFinder^TM^ COVID-19 PLUS RealAmp assay (OSANG Healthcare Co., Ltd, Anyang-si, Korea) for qualitative virus detection through reverse transcription and real-time polymerase chain reaction from RNA.

### 2.4. DNA Extraction and Amplification

The feasibility of using NPS collected in viral transport media (VTM) for SARS-CoV-2 detection and microbiota analysis has been previously reported [17]. The isolation of total nucleic acid from the NPS-VTM samples was performed in the Automated ELITe InGenius Platform using the ELITe InGenius ^TM^ SP 200 extraction kit following the manufacturer’s protocol (ELITechGroup S.p.A., Turin, Italy).

The DNA concentration in the samples was estimated using a Qubit fluorometer (Invitrogen, Thermo Fisher Scientific Inc, Waltham, MA, USA). The samples were subjected to amplification of the V4 region of the 16S rRNA gene using the V4 specific primer set 515F/806R [18]. Briefly, triplicate PCR amplifications of each sample were prepared in 25 μL reaction volumes using the QIAGEN PCR kit, containing between 12.9 and 14.9 μL of molecular water, 2.25 μL of 10X Buffer, 0.7 μL of 25 mM MgCl_2_, 1 μL of dNTP 10 mM each, 2.25 μL of Q solution 5X, 1.25 μL primers 515F and 806R, 0.4 μL of Taq DNA polymerase, and 30 ng of DNA template (1 to 3 μL). The reaction conditions were as follows: denaturation at 94 °C for 3 min followed by 35 cycles of denaturation at 94 °C for 45 s, annealing at 50 °C for 1 min, elongation at 72 °C for 1.5 min, and final elongation at 72 °C for 10 min. The PCR product was verified through 1.5% agarose gel electrophoresis with 3 μL of the sample.

### 2.5. Library Preparation

The DNA library preparation and amplicon sequencing was performed as in Quintero et al. 2022 [19]. Briefly, the PCR amplicon triplicates were combined for each sample and subjected to a second PCR to incorporate barcode indexes and Illumina adapters in a 25 µL reaction (including 14.75 µL of DNase-free water, 0.25 µL of Taq PCR Master Mix (Qiagen, Valencia, CA, USA), 1 µL of each index primer (forward and reverse), and 2 µL of pooled PCR product). The PCR settings included denaturation at 94 °C for 3 min, followed by 35 cycles of denaturation at 94 °C, for 45 min, hybridization at 50 °C for 1 min, elongation at 72 °C for 1 min 30 s, and a final elongation of 10 min at 72 °C. The resulting amplicons from the second PCR were then purified using AMPURE XP paramagnetic beads (Beckman Coulter, Indianapolis, IN, USA) in a volume of 5 µL. The DNA library’s concentration and quality were determined using a Qubit fluorometer (Turner BioSystems, Foster City, CA, USA) and a 2100 Bioanalyzer instrument (Agilent, Santa Clara, CA, USA). Amplicon sequencing was performed on an Illumina MiSeq System (Illumina, San Diego, CA, USA) to generate 2 × 250 bp paired-end reads in the Naos Molecular Laboratories of the Smithsonian Tropical Research Institute (STRI, Panama, Panama). The sequence reads generated in this study were deposited in the NCBI Sequence Read Archive (SRA) under Bioproject PRJNA1132178.

### 2.6. Data Analyses

We used the Quantitative Insights into Microbial Ecology 2 (QIIME 2.0) [20] platform to analyze the 16S rRNA sequences generated from the NPS-VTM samples. The sequences were then quality-filtered and denoised using the DADA2 algorithm [21] within the QIIME2 (v2023.2) workflow. Amplicon sequence variants (ASVs) were generated and used for all downstream analyses. Finally, the ASVs were classified from the kingdom to the genus rank using the Silva reference 16S rRNA gene database, version 138.1, resulting in the construction of an ASV table with the read counts of all ASVs in all samples. The ASVs classified as chloroplasts or mitochondria and those with less than 10 counts were removed from the dataset.

To explore the naso-oropharyngeal microbiota data, a phyloseq object was generated using the phyloseq R package (v1.42.0) [22]. Alpha diversity was estimated using Faith’s phylogenetic diversity (Faith’s PD), evenness, observed features, and Shannon indices, followed by non-parametric Kruskal–Wallis to examine the statistical differences among patients with detected and not-detected SARS-CoV-2, age categories, and patient status groups.

For the ordination and visualization of the taxonomic composition (beta diversity), PCoA was performed based on the weighted UniFrac distance method using the phyloseq and ggplot2 (v3.5.1) packages [22]. The statistical significances among the SARS-CoV-2 test result, age, and patient status were assessed using Adonis test analyses with 999 permutations for each analysis in the vegan R package (v2.6-4).

To identify candidate biomarkers in the SARS-CoV-2 test result and patient status groups, a linear discriminant analysis effect size (LEfSe) analysis was conducted using the web platform MicrobiomeAnalyst 2.0, with the following parameters: LDA score > 2, *p* < 0.05 (https://www.microbiomeanalyst.ca/MicrobiomeAnalyst/upload/OtuUploadView.xhtml) (accessed on 4 January 2024) [23].

Phylogenetic Investigation of Communities by Reconstruction of Unobserved States (PICRUSt2) v2.5.2 [24] was used to predict the bacterial functional profile in the SARS-CoV-2 test result groups based on the 16S rRNA gene data. Differential abundance analyses of the inferred functions were conducted using ggpicrust2 R package v1.7.3 [25].

## 3. Results

### 3.1. Alpha and Beta Diversity

**Figure 1:**
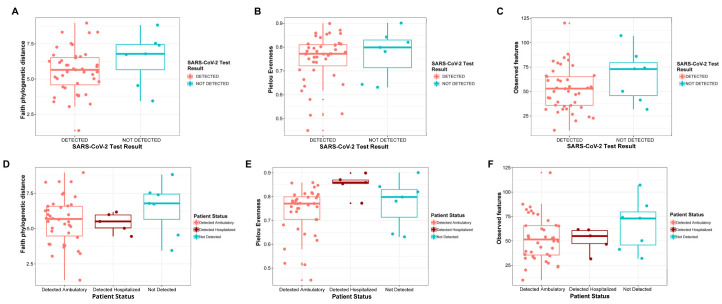
Alpha diversity of naso-oropharyngeal bacterial communities in patients with suspected SARS-CoV-2 in Panama. Alpha diversity was estimated using Faith’s phylogenetic diversity (Faith’s PD), Pielou evenness, and observed features according to (**A**–**C**) SARS-CoV-2 test results (detected and not detected) and (**D**–**F**) patient status (detected ambulatory, detected hospitalized, and not detected).

The NPS microbiota of individuals with detected SARS-CoV-2 (n = 43) and not-detected (n = 7) SARS-CoV-2 was evaluated through 16S rRNA gene amplicon sequencing. The alpha diversity measures of NPS were calculated to estimate the bacterial 16S rRNA gene diversity in this niche. The Kruskal–Wallis test was used to examine the statistical differences between the two groups, and no significant differences (*p* > 0.05) between individuals with detected SARS-CoV-2 and not-detected SARS-CoV-2 were found when evaluating Faith’s PD, evenness, observed features, and Shannon indices (Figure 1A–C and Figure S1, Table S2). Additionally, the same diversity indices were calculated to estimate the alpha diversity by patient status (Figure 1D–F and Figure S1, Table S2), followed by the non-parametric Kruskal–Wallis test. According to these results, there were significant differences among patient statuses (*p* = 0.0109) for the Pielou evenness measure (Figure 1E, Table S2) but not for the other alpha diversity indices. The alpha diversity parameters did not show significant differences between age categories (Figure S2, Table S2).

**Figure 2:**
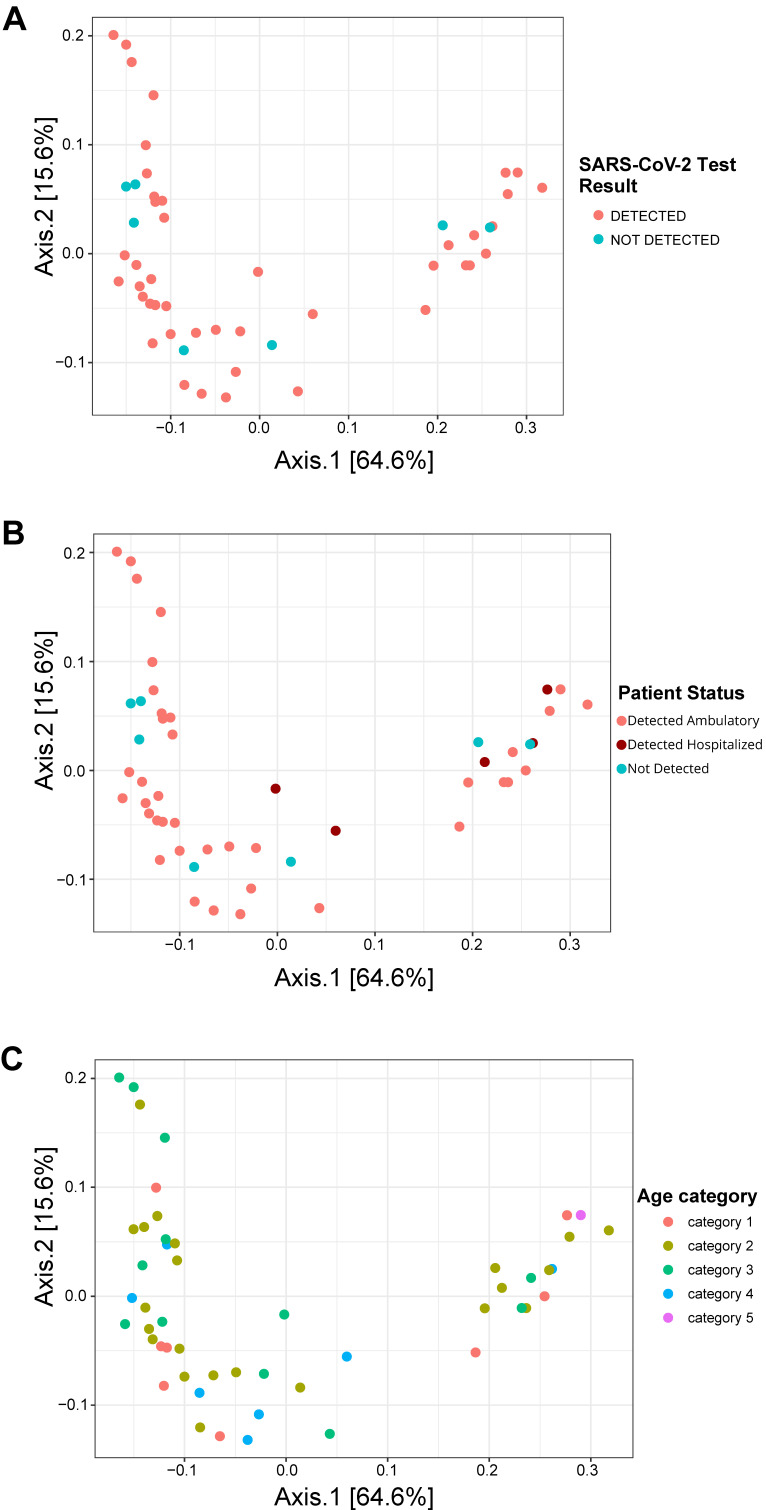
Beta diversity of naso-oropharyngeal bacterial communities in patients with suspected SARS-CoV-2 in Panama. Ordination plots derived from principal coordinates analysis (PCoA) showing the overall composition of the microbial community in the (**A**) SARS-CoV-2 test results, (**B**) patient status, and (**C**) age category. PCoA was performed using the weighted UniFrac distance method. Age categories: Category 1 (less than 20 years old), Category 2 (20–39 years old), Category 3 (40–59 years old), Category 4 (60–80 years old), and Category 5 (more than 80 years old).

No significant differences were observed in the naso-oropharyngeal microbial community between patients with detected and non-detected SARS-CoV-2 in the beta diversity analyses (*p* = 0.839) for the SARS-CoV-2 test results (Figure 2A, Table S3). Additionally, according to the statistical analysis (Adonis test), there were no significant differences in the microbial communities when compared by patient status (*p* = 0.269) or age category (*p* = 0.319) (Figure 2B and Figure 2C, respectively).

### 3.2. Relative Abundance

**Figure 3:**
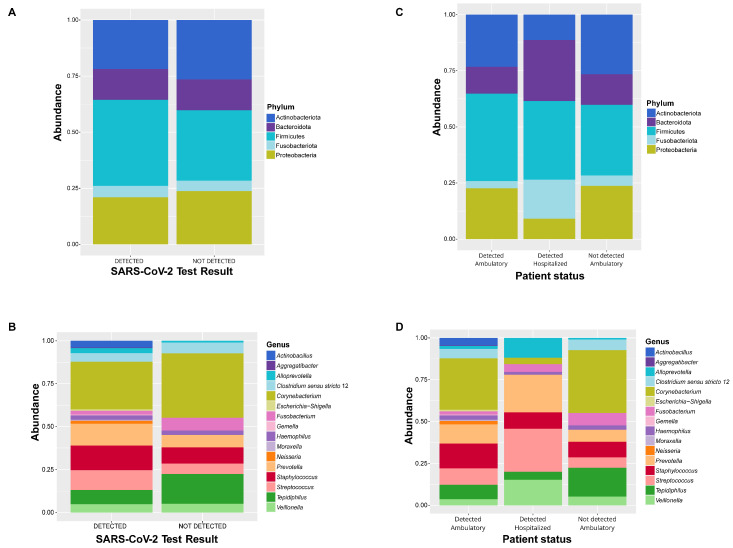
Bacterial profiles of patients with suspected SARS-CoV-2 (**A**,**B**) SARS-CoV-2 test results: detected and not-detected patients. (**C**,**D**) Patient status: detected ambulatory, detected hospitalized, and not detected. Bar plots show the relative abundance of dominant bacterial taxa at phylum and genus levels.

The microbial communities in both groups of SARS-CoV-2 test results: detected (D) and not detected (ND) were dominated by the phyla Firmicutes (D: 36.24%, ND: 27.02%), Actinobacteriota (D: 26.82%, ND: 35.57%), Proteobacteria (D: 19.20%, ND: 20.89%), Bacteroidota (D: 15.31%, ND: 9.17%), and Fusobacteriota (D: 2.43%, ND: 7.35%) (Figure 3A, Table S4). At the family level, the most dominant were *Corynebacteriaceae* (D: 26.82%, ND: 35.57%), *Prevotellaceae* (D: 15.31%, ND: 9.17%), *Staphylococcaceae* (D: 14.02%, ND: 8.86%), *Streptococcaceae* (D: 11.63%, ND: 5.77%), and *Hydrogenophilaceae* (D: 8.19%, ND: 16.60%) (Figure S3 and Table S4). Moreover, the most abundant genera were *Corynebacterium* (D: 26.82%, ND: 35.57%), *Staphylococcus* (D: 14.02%, ND: 8.86%), *Prevotella* (D: 12.51%, ND: 7.63%), *Streptococcus* (D: 11.63%, ND: 5.77%), and *Tepidiphilus* (D: 8.19%, ND: 16.60%) (Figure 3B, Table S4).

Regarding to the patient status, Firmicutes was the most dominant phylum in the detected ambulatory (34.46%) and detected hospitalized (49.72%) groups, while Actinobacteriota (35.57%) was the most prevalent phylum in the not-detected group (Figure 3C, Table S5). At the family level, the most abundant were *Corynebacteriaceae* in the detected ambulatory and not-detected groups (29.83% and 35.57%, respectively) and *Prevotellaceae* in the detected hospitalized group (32.01%) (Figure S3, Table S5). At the genus level, *Corynebacterium* was the most abundant genus for both detected ambulatory (29.83%) and not detected (35.57%), followed by *Staphylococcus* (DA: 14.62%, DH: 9.47%, ND: 8.86%), *Prevotella* (DA: 11.40%, DH: 20.95%, ND: 7.63%), and *Streptococcus* (DA: 9.88%, DH: 25%, ND: 5.77%) (Figure 3D, Table S5).

### 3.3. Linear Discriminant Analysis Effect Size (LEfSe)

**Figure 4:**
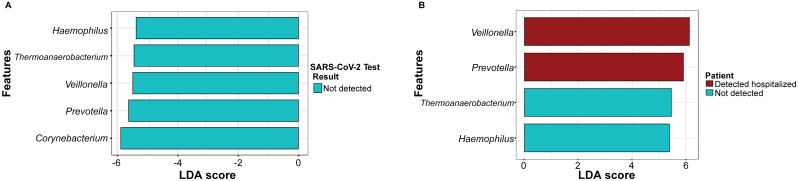
Biomarker exploration in the (**A**) SARS-CoV-2 test results for detected and not detected patients at genus level and (**B**) patient status. Linear discriminant analysis (LDA) scores were generated using LDA effect size (LEfSe) analysis.

LEfSe analysis was conducted to identify candidate biomarkers in the SARS-CoV-2 test result and patient status groups (Figure 4, Supplementary Material: Tables S6 and S7). For the SARS-CoV-2 test result groups (Figure 4A, Table S6), significant differences were found among the sample groups in five genera: *Corynebacterium*, *Prevotella*, *Veillonella*, *Thermoanaerobacterium*, and *Haemophilus* in the not-detected group, while no candidate biomarkers were found for the detected group. For the patient status groups, significant differences were found in four genera: *Prevotella* and *Veillonella* (detected hospitalized), *Haemophilus and Thermoanaerobacterium* (not detected) (Figure 4B, Table S7). No significantly different genera were found in the detected ambulatory group.

### 3.4. Bacterial Functional Analysis in the SARS-CoV-2 Test Result Groups

The functional profiles of the naso-oropharyngeal microbiota in the SARS-CoV-2 test result groups were analyzed using PICRUST2, based on the 16S rRNA data. A total of 18 significantly enriched functional pathways were predicted. The enrichment of seven Kyoto Encyclopedia of Genes and Genomes (KEGG) pathways was observed in the detected group, which are involved in xenobiotic degradation and metabolism, metabolism of terpenoids and polyketides, amino acid metabolism, human diseases, and environmental information processing. In contrast, 11 metabolic pathways were enriched in the not-detected group; these pathways were mainly represented by cellular processes, glycan biosynthesis, and lipid metabolism (Figure S4).

## 4. Discussion

In this study, the SARS-CoV-2 infection was not associated with significant changes in the naso-oropharyngeal bacterial community at the time of the COVID-19 diagnosis. Our findings suggest that there were no significant changes in the bacterial diversity in response to SARS-CoV-2 or that the virus was unable to induce these changes in the patients, according to the alpha and beta diversity analyses for SARS-CoV-2 test results. This observation aligns with previous reports made on COVID-19 suspected cases, such as Braun et al. 2021 [26], where no significant effect of SARS-CoV-2 on the nasopharyngeal microbial community was observed using multiple analysis methods in positive subjects in comparison with SARS-CoV-2-negative subjects. Similarly, other studies on naso-oropharyngeal swabs showed no significant difference in the microbiota of patients with suspected COVID-19 who tested positive and those who tested negative [2,27]. These findings support that the naso-oropharyngeal microbiota may be less affected by SARS-CoV-2, even when dysbiosis of the naso-oropharyngeal microbiota is associated with respiratory diseases [2]. Our results showed that the bacterial community structure had significant changes when comparing patient status (detected ambulatory, detected hospitalized, and not detected) when Pielou’s evenness diversity measure was evaluated. This agrees with previous studies on patients with COVID-19 with different disease severities in which significant differences were found between the mild and severe cohorts [12].

The microbial communities in both groups (patients with detected and not-detected SARS-CoV-2) were dominated by Proteobacteria, Actinobacteriota, Bacteroidota, and Fusobacteriota, which aligned with prior research suggesting that the naso-oropharyngeal microbiota of patients with suspected COVID-19, positive and negative, belonged to these bacterial phyla [2,10,27].

According to the patient status, Firmicutes was the most dominant phylum in the detected ambulatory and detected hospitalized groups, followed by Actinobacteriota and Bacteroidota, while Actinobacteriota and Firmicutes were the most common taxa in the not-detected group. This is consistent with earlier research in which similar bacterial taxa have been reported in oropharyngeal and nasopharyngeal samples from patients with different COVID-19 disease severities [13,28].

Our results showed that SARS-CoV-2 does not affect the overall composition of the microbiome but causes changes in the abundance of certain taxa at the genus level, such as a decrease in *Corynebacterium* and an increase in *Staphylococcus*, *Prevotella,* and *Streptococcus* in patients with detected COVID-19. These findings are consistent with previous reports that the nasal commensal organism *Corynebacterium* (Actinobacteria) showed a decreased relative abundance in patients with COVID-19 [10,29]. *Streptococcus* and *Staphylococcus* are not only commonly found in the upper respiratory environments as commensal bacterial genera but also as opportunistic pathogens [7,30]. Although the results regarding the relative abundance of *Staphylococcus* and *Streptococcus* in patients with suspected COVID-19 vary, both genera have been reported to be the predominant taxa in the naso-oropharyngeal microbiota of such patients [10,27]. The genus *Prevotella*, commensal bacteria in the human oral microbiota, has been reported as a common genus in patients with COVID-19 who developed the most severe disease [31]. *Prevotella* has also been reported as part of the nasopharyngeal microbiota profile of pregnant women infected with SARS-CoV-2 [32].

Previous studies have suggested that certain bacterial taxa in the naso-oropharyngeal microbiota are related to COVID-19 infection and that these taxa could potentially serve as diagnostic biomarkers to distinguish patients with detected COVID-19 from those with non-detected COVID-19 [2,12]. A linear discriminant analysis Effect Size (LEfSe) analysis was conducted to identify candidate biomarkers in the SARS-CoV-2 test result and patient status groups. Our results revealed significant differences in the SARS-CoV-2 test results for *Corynebacterium*, *Prevotella*, *Veillonella*, *Thermoanaerobacterium*, and *Haemophilus* in the not-detected group. There were significant differences in the abundance of the taxa according to the patient status: in particular, for *Prevotella* and *Veillonella* in detected hospitalized patients and for *Haemophilus* and *Thermoanaerobacterium* in not-detected patients.

The genus *Corynebacterium* is a common member of the human URT microbiota, specifically in the naso-oropharyngeal region, since early life [7,33]. A previous study suggested a protective role of *Corynebacterium* against respiratory tract pathogenic bacteria such as *S. aureus* and *S. pneumoniae*, which are the most common cause of post-influenza pneumonia [30,34]. Another study revealed that nasopharyngeal microbiota composition changes were associated with the severity of the disease; in particular, *Corynebacterium* consistently decreased as COVID-19 severity increased [11]. The decrease in the relative abundance of *Corynebacterium* in detected patients in this study confirms the relevance of this genus as a potential biomarker during a viral infection such as SARS-CoV-2.

In previous studies, the bacterial genera *Prevotella* and *Veillonella* have been found to be dominant taxa in both SARS-CoV-2-infected and non-infected individuals [8]. *Prevotella* and *Veillonella* have been reported as the top bacterial genera enriched in the naso-oropharyngeal microbiota of patients with and without COVID-19 [2]. *Prevotella* was found to be positively associated with SARS-CoV-2 severity in the nasopharyngeal microbiota [35] as is the case with our study. *Thermoanaerobacterium* has been reported as part of the microbiota of the URT, as demonstrated by Rueca et al. in 2021 [36] detected in naso-oropharyngeal swabs of healthy subjects. However, the low abundance of this genus has been reported in SARS-CoV-2-asymptomatic/paucisymptomatic subjects [37]. Our findings revealed *Haemophilus* as a taxon enriched in patients who were not detected for SARS-CoV-2. This genus has been reported to show a decrease in abundance in patients with COVID but has not been previously identified as a possible biomarker [8]. *Haemophilus* has been found to be enriched in patients with viral respiratory illnesses alongside pathogens such as *Streptococcus* and *Moraxella* [38]. This enrichment in patients who were not detected may suggest symptom similarities with COVID-19, potentially indicating involvement in other respiratory diseases.

To obtain functional insight between the SARS-CoV-2 tests result groups (detected and not detected), we predicted the metabolic functions of the naso-oropharyngeal microbiota using PICRUST2 [24]. In the detected group, results revealed the significant enrichment of xenobiotic degradation and metabolism, including benzoate and aminobenzoate degradation. Similarly, these metabolic pathways were also observed to be significantly enriched in patients with COVID-19 based on shotgun metagenomic studies of oropharyngeal swabs [39]. Metabolic pathways associated with environmental information processing (neuroactive ligand–receptor interaction) and neurodegenerative diseases were enriched in subjects with detected SARS-CoV-2. This suggests that the microbiota may play a role in SARS-CoV-2 neuroinvasion. The virus binds ACE2 receptors, leading to inflammation and disrupting the URT microbiota, which has been implicated in neurological diseases [4,40,41]. Flagellar assembly and bacterial chemotaxis pathways were significantly enriched in the detected and not-detected SARS-CoV-2 test result groups, respectively. These pathways have been observed in the microbiota of influenza A virus-positive patients, showing an increased expression of genes related to cell motility and signal transduction [42,43]. Furthermore, the flagellar assembly pathways were also enriched in URT microbiota of children who tested positive for COVID-19 [44].

## 5. Conclusions

This study provides information on the bacterial community composition of the naso-oropharynx in the respiratory tract of suspected SARS-CoV-2 cases from Veraguas province in Panama. SARS-CoV-2 did not alter the overall microbiota composition but influenced the abundance of specific taxa at the genus rank. Specifically, a decrease in the abundance of *Corynebacterium* in patients with detected SARS-CoV-2. *Veillonella, Prevotella, Thermoanaerobacterium,* and *Haemophilus* were identified as potential biomarkers to distinguish patients with detected SARS-CoV-2 and hospitalized patients from patients with non-detected SARS-CoV-2. Further investigation is necessary to better understand the importance of the naso-oropharyngeal microbiota as a diagnostic biomarker for SARS-CoV-2, as it provides insights into the diversity, composition, and resilience of the microbial community in patients with SARS-CoV-2.

## Data Availability

The raw data (DNA sequences) supporting the conclusions of this article are available at the NCBI Sequence Read Archive (SRA) under Bioproject PRJNA1132178. https://www.ncbi.nlm.nih.gov/sra/PRJNA1132178.

## Supplementary Materials

The following supporting information can be downloaded at https://www.mdpi.com/article/10.3390/pathogens13080615/s1: Table S1. Study subjects’ characteristics; Table S2. Alpha diversity statistical values in naso-oropharyngeal samples; Table S3. Beta diversity statistical values in naso-oropharyngeal samples; Table S4. Relative abundance of the most common bacterial taxa in patients with detected and not-detected SARS-CoV-2; Table S5. Relative abundance of the most common bacterial taxa according to patient status; Table S6. LEfSe analysis* for SARS-CoV-2 test results at different taxonomic levels.; Table S7. LEfSe analysis* for severity group at different taxonomic levels; Figure S1. Alpha diversity of naso-oropharyngeal bacterial communities in patients with suspected SARS-CoV-2 in Panama. Alpha diversity was estimated using Shannon biodiversity indices according to (A) SARS-CoV-2 test results (detected and not detected), and (B) patient status (detected ambulatory, detected hospitalized, and not detected); Figure S2. Alpha diversity of naso-oropharyngeal bacterial communities in patients with suspected SARS-CoV-2 in Panama according to age category. Alpha diversity was estimated using (A) Faith’s phylogenetic diversity (Faith’s PD), (B) Pielou evenness, (C) observed features, and (D) Shannon diversity indices. The center line of each box plot represents the median, the lower and upper hinges represent the first and third quartiles, and the whiskers represent + 1.5 the interquartile range. Age categories: Category 1 (less than 20 years old), Category 2 (20–39 years old), Category 3 (40–59 years old), Category 4 (60–80 years old), and Category 5 (more than 80 years old); Figure S3. Microbial profiles of patients with suspected SARS-CoV-2 (A) SARS-CoV-2 test results: detected and not detected patients. (B) Patient status: detected ambulatory, detected hospitalized, and not detected. Bar plots show the relative abundance of dominant bacterial taxa at family level. Figure S4. Prediction of metabolic pathways enriched in detected and not-detected suspected patients of SARS-CoV-2. PICRUST2 was used to predict the bacterial functional profile in the SARS-CoV-2 test result groups based on 16S rRNA gene data. KEGG pathways are categorized by the main pathway class. The bar plots illustrate the relative abundance of each KEGG pathway and the log2 fold change, expression for each pathway across the two groups.
