# Supplementary material for: Insights into the Naso-Oropharyngeal Bacterial Composition in Suspected SARS-CoV-2 Cases
Source: Pathogens. 2024 Jul 25;13(8):615. doi: 10.3390/pathogens13080615 (PMC11357247; doi:10.3390/pathogens13080615)
Supplement: Supplementary file 1 [file pathogens-13-00615-s001.zip › pathogens-3056899-supplementary.pdf]

Supplementary figures

Article

Insights into the naso-oropharyngeal bacterial composition in suspected SARS-CoV-2 cases.

Librada A. Atencio[1], Indira J. Quintero[1], Alejandro Almanza[1], Gilberto Eskildsen[2], Joel Sánchez-Gallego[3,4], Mellissa Herrera[5], Hermógenes Fernández-Marín[1], José R. Loaiza[1,6,*] and Luis C. Mejía[1,6,7,*]

[1] Centro de Biodiversidad y Descubrimiento de Drogas, Instituto de Investigaciones Científicas y Servicios de Alta Tecnología (INDICASAT), Clayton, Panama City 0843-01103, Panama; latencio@indicasat.org.pa (L.A.A), indiravarg@gmail.com (I.J.Q), aalmanza@indicasat.org.pa (A.A), hfernandez@indicasat.org.pa (H.F.M).
[2] Departamento de Microbiología Humana, Facultad de Medicina, Universidad de Panamá; gilberto.eskildsen@up.ac.pa (G.E.).
[3] Department of Marine Earth and Atmospheric Sciences, North Carolina State University, Raleigh, North Carolina 27695-8208, United States of America; jjsanche@ncsu.edu (J.S.G.).
[4] Coiba Scientific Station (COIBA AIP), Gustavo Lara Street, Bld. 145B, City of Knowledge, Clayton, Panama City 0843-01853, Panama.
[5] Hospital Luis "Chicho" Fábrega, MINSA, Veraguas, Panamá; myherrera@minsa.gob.pa (M.H.).
[6] Smithsonian Tropical Research Institute, Panamá 0843-03092, Panama
[7] Departamento de Genética y Biología Molecular, Universidad de Panamá, Panamá, Estafeta Universitaria Apartado 3366, Zona 4, Panamá.
* Correspondence: jloaiza@indicasat.org.pa (J.R.L.), lmejia@indicasat.org.pa (L.C.M.); Tel.: +507-517-0700.

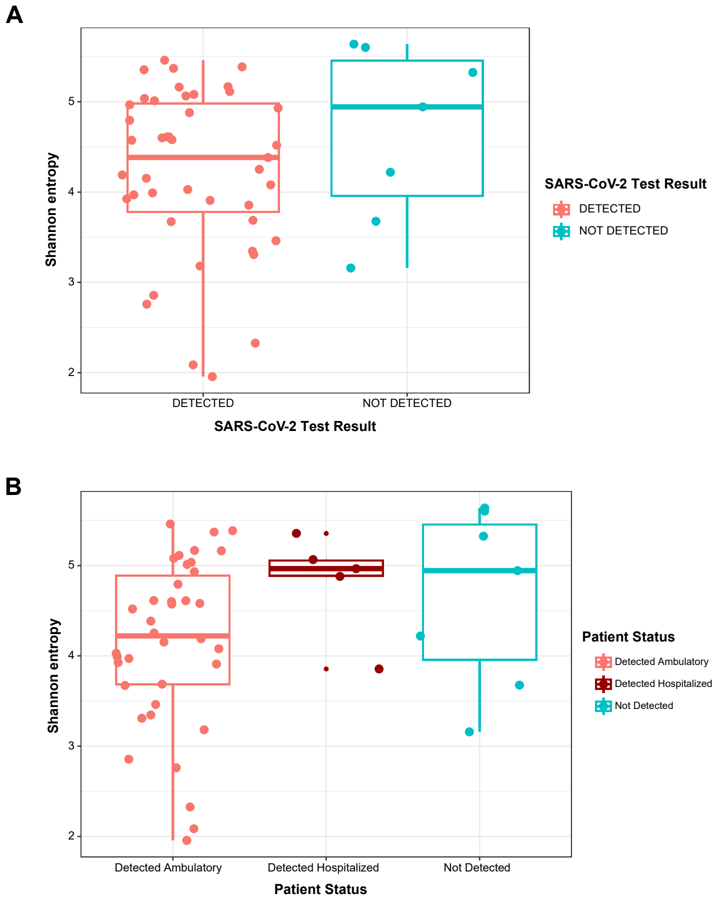

Figure S1. Alpha diversity of naso-oropharyngeal bacterial communities in suspected SARS-CoV-2 patients in Panama. Alpha diversity was estimated using Shannon biodiversity indice according to A) SARS-CoV-2 test results (detected and not detected), and B) Patienten status (detected ambulatory, detected hospitalized and not detected).

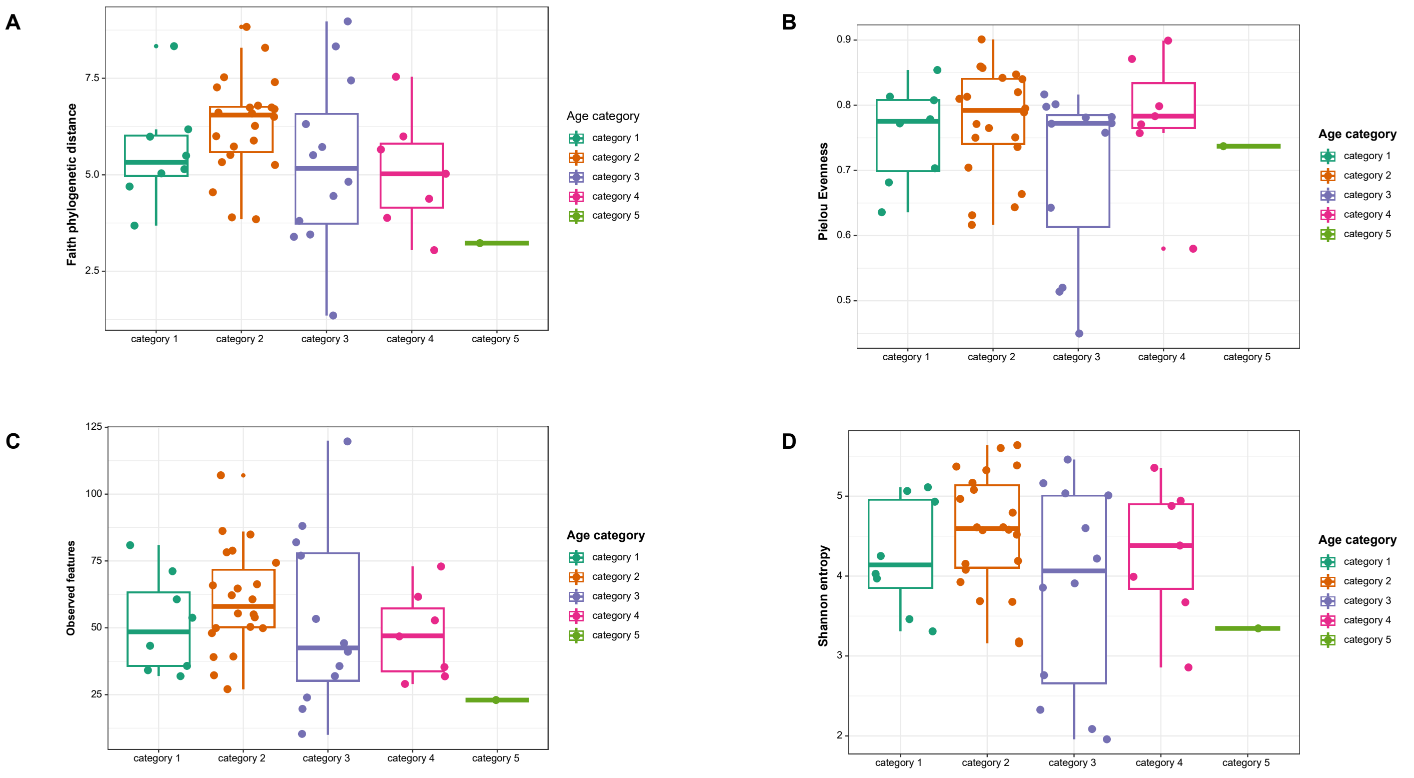

Figure S2. Alpha diversity of naso-oropharyngeal bacterial communities in suspected SARS-CoV-2 patients in Panama according to age category. Alpha diversity was estimated using A) Faith's phylogenetic diversity (Faith's PD), B) Pielou evenness, C) observed features and D) Shannon diversity indices. The center line of each box plot represents the median, the lower and upper hinges represent the first and third quartiles and whiskers represent + 1.5 the interquartile range. Age categories: Category 1 (less than 20 years old), Category 2 (20-39 years old), Category 3 (40-59 years old), Category 4 (60-80 years old) and Category 5 (more than 80 years old).

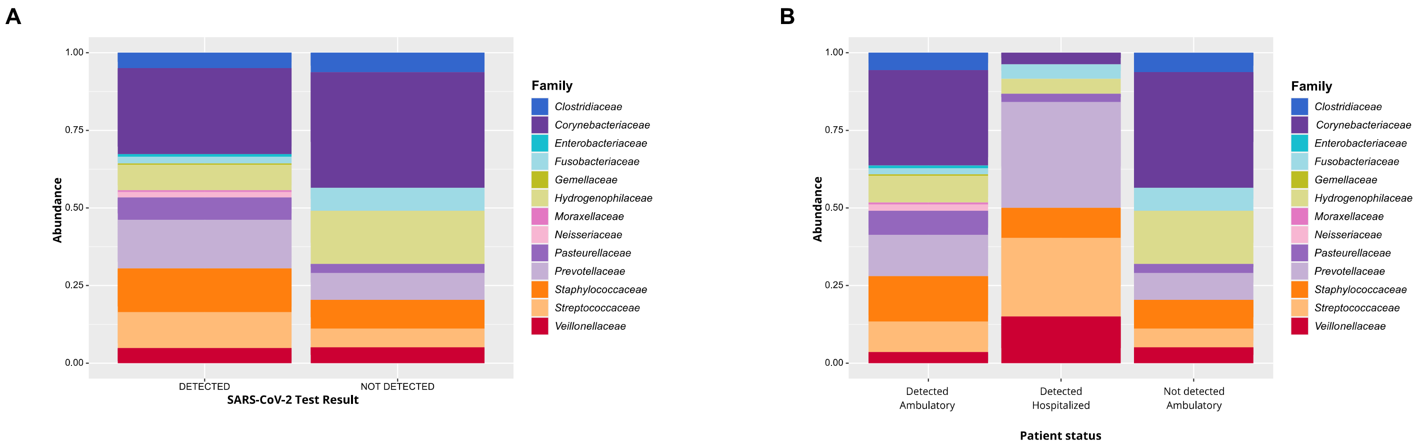

Figure S3. Microbial profiles of suspected SARS-CoV-2 patients A) SARS-CoV-2 test results, detected, and not detected patients. B) Patient status, detected ambulatory, detected hospitalized and not detected. Bar plots show the relative abundance of dominant bacterial taxa at family level.

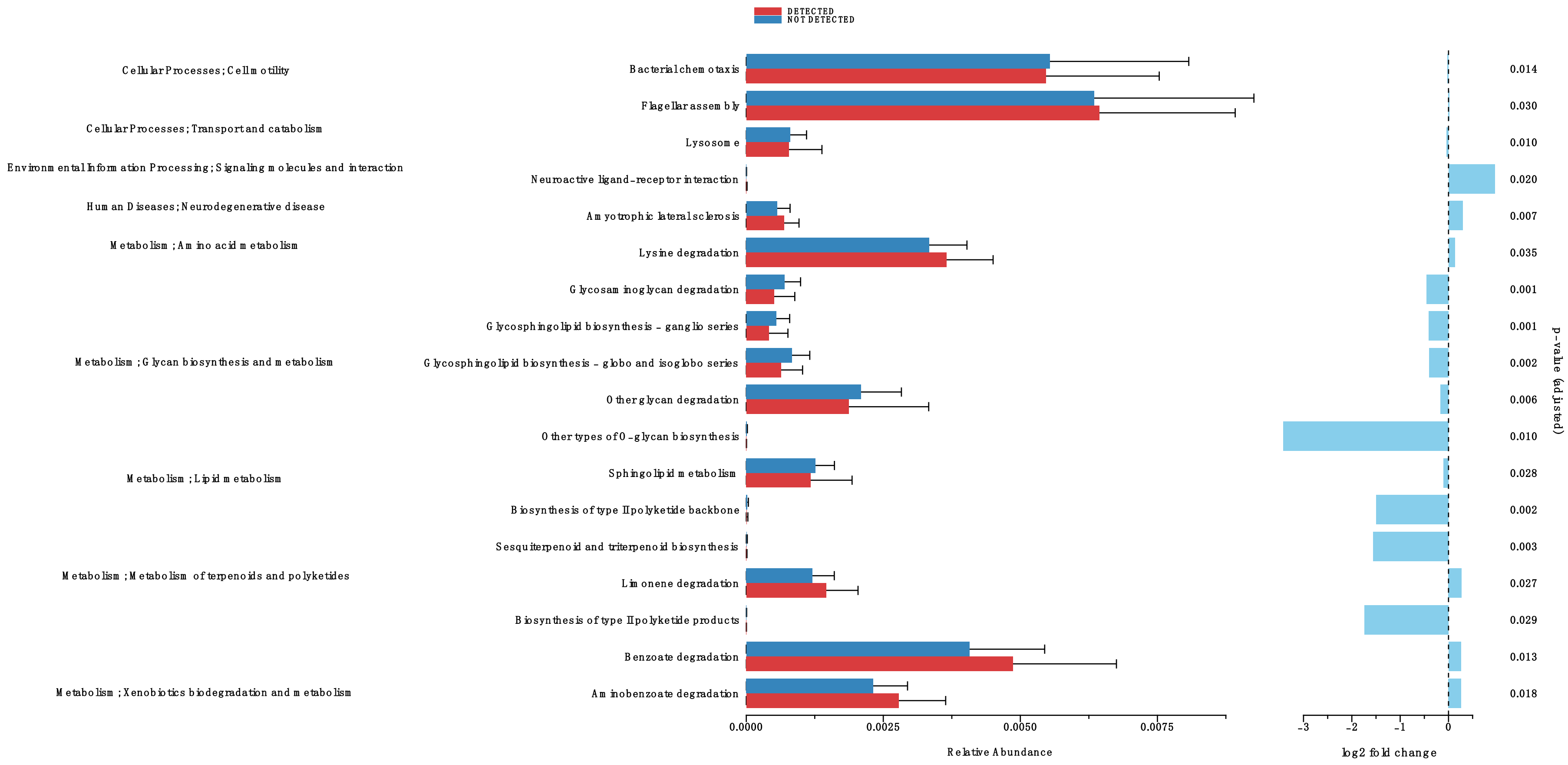

Figure S4. Prediction of metabolic pathways enriched in detected and not-detected suspected patients of SARS-CoV-2. PICRUST2 was used to predict the bacterial functional profile in the SARS-CoV-2 test results groups based on 16S rRNA gene data. KEGG pathways are categorized by the main pathway class. The bar plots illustrate the relative abundance of each KEGG pathway, and the log2 fold change, expression for each pathway across the two groups.

Table S1. Study subjects characteristics

	Characteristic	Detected (n=43)	Not detected (n=7)
Patient status	Detected-Ambulatory	38	0
	Detected-Hospitalized	5	0
	Not detected	0	7
Age category	category 1 (less than 20 years old)	8	0
	category 2 (20-39 years old)	17	5
	category 3 (40-59 years old)	11	1
	category 4 (60-80 years old)	6	1
	category 5 (more than 80 years old)	1	0
Gender	Female	23	4
	Male	20	3

Table S2. Alpha Diversity statistical values in naso-oropharyngeal samples.

FAITH PHYLOGENETIC DISTANCE			
**Krustal-Wallis test**	**SARS-cov-2 test results**	**Age category**	**Patient status**
Kruskal-Wallis chi-squared	2.2374	8.0524	2.35
p-value	0.1347	0.08968	0.3088

EVENNESS			
**Krustal-Wallis test**	**SARS-cov-2 test results**	**Age category**	**Patient status**
Kruskal-Wallis chi-squared	0.6349	3.3204	9.0357
p-value	0.4255	0.5057	0.0109

OBSERVED FEATURES			
**Krustal-Wallis test**	**SARS-cov-2 test results**	**Age category**	**Patient status**
Kruskal-Wallis chi-squared	1.5846	5.7785	1.5852
p-value	0.2081	0.2163	0.4527

SHANNON			
**Krustal-Wallis test**	**SARS-cov-2 test results**	**Age category**	**Patient status**
Kruskal-Wallis chi-squared	1.2822	3.9201	3.3637
p-value	0.2575	0.4169	0.1860

Table S3. Beta Diversity statistical values in naso-oropharyngeal samples

**Adonis test***	**SARS-cov-2 test results**	**Age category**	**Patient Status**
ANOSIM statistic R	-0.08041	0.01784	0.04096
Significance	**0.839**	**0.319**	**0.269**

*Number of permutations: 999

Adonis test results are based on a weighted unifrac distance matrix.

Table S4. Relative abundance of the most common bacterial taxa in SARS-CoV-2 detected and not detected patients

Taxonomic level	Detected	Not detected
	Mean RA % ± (SD) %	Mean RA % ± (SD) %
**Phylum**		
Firmicutes	36.24 ± 24.71	27.02 ± 15.7
Actinobacteriota	26.82 ± 32.37	35.57 ± 35.68
Proteobacteria	19.2 ± 23.04	20.89 ± 15.86
Bacteroidota	15.31 ± 25.1	9.17 ± 14.43
Fusobacteriota	2.43 ± 4.44	7.35 ± 10.27
**Family**		
Corynebacteriaceae	26.82 ± 32.37	35.57 ± 35.68
Prevotellaceae	15.31 ± 25.1	9.17 ± 14.43
Staphylococcaceae	14.02 ± 20.67	8.86 ± 10.24
Streptococcaceae	11.63 ± 15.48	5.77 ± 6.02
Hydrogenophilaceae	8.19 ± 16.6	16.6 ± 17.7
Pasteurellaceae	7.6 ± 16.55	4 ± 4.74
Clostridiaceae	4.89 ± 14.06	6.04 ± 6.16
Veillonellaceae	4.89 ± 7.99	5.08 ± 8.19
Fusobacteriaceae	2.43 ± 4.44	7.35 ± 10.27
Neisseriaceae	1.91 ± 6.09	0.23 ± 0.62
Enterobacteriaceae	0.87 ± 5.19	0.06 ± 0.15
Gemellaceae	0.81 ± 2.79	1.26 ± 1.69
Moraxellaceae	0.63 ± 2.85	0 ± 0
**Genus**		
Corynebacterium	26.82 ± 32.37	35.57 ± 35.68
Staphylococcus	14.02 ± 20.67	8.86 ± 10.24
Prevotella	12.51 ± 21.77	7.63 ± 11.95
Streptococcus	11.63 ± 15.48	5.77 ± 6.02
Tepidiphilus	8.19 ± 16.6	16.6 ± 17.7
Clostridium sensu stricto 12	4.89 ± 14.06	6.04 ± 6.16
Veillonella	4.89 ± 7.99	5.08 ± 8.19
Actinobacillus	3.91 ± 15.99	0.39 ± 1.03
Haemophilus	3.32 ± 5.45	3.61 ± 3.88
Alloprevotella	2.8 ± 6.22	1.54 ± 2.49
Fusobacterium	2.43 ± 4.44	7.35 ± 10.27
Neisseria	1.91 ± 6.09	0.23 ± 0.62
Escherichia-Shigella	0.87 ± 5.19	0.06 ± 0.15
Gemella	0.81 ± 2.79	1.26 ± 1.69
Moraxella	0.63 ± 2.85	0 ± 0
Aggregatibacter	0.38 ± 1.57	0 ± 0

Table S5. Relative abundance of the most common bacterial taxa according to patient status.

Taxonomic level	Detected Ambulatory	Detected Hospitalized	Not detected
	Mean RA % ± (SD) %	Mean RA % ± (SD) %	Mean RA % ± (SD) %
**Phylum**			
Firmicutes	34.46 ± 23.45	49.72 ± 32.71	27.02 ± 15.7
Actinobacteriota	29.83 ± 33.28	3.97 ± 4.57	35.57 ± 35.68
Proteobacteria	20.47 ± 23.84	9.58 ± 13.52	20.89 ± 15.86
Bacteroidota	13.11 ± 24.31	32.01 ± 27.46	9.17 ± 14.43
Fusobacteriota	2.13 ± 4.24	4.72 ± 5.76	7.35 ± 10.27
**Family**			
Corynebacteriaceae	29.83 ± 33.28	3.97 ± 4.57	35.57 ± 35.68
Staphylococcaceae	14.62 ± 20.91	9.47 ± 20.28	8.86 ± 10.24
Prevotellaceae	13.11 ± 24.31	32.01 ± 27.46	9.17 ± 14.43
Streptococcaceae	9.88 ± 14.11	25 ± 20.53	5.77 ± 6.02
Hydrogenophilaceae	8.6 ± 17.34	5.08 ± 9.8	16.6 ± 17.7
Pasteurellaceae	8.17 ± 17.53	3.34 ± 2.99	4 ± 4.74
Clostridiaceae	5.51 ± 14.87	0.19 ± 0.44	6.04 ± 6.16
Veillonellaceae	3.67 ± 7.19	14.11 ± 8.48	5.08 ± 8.19
Fusobacteriaceae	2.13 ± 4.24	4.72 ± 5.76	7.35 ± 10.27
Neisseriaceae	2.1 ± 6.46	0.51 ± 1.13	0.23 ± 0.62
Enterobacteriaceae	0.89 ± 5.51	0.66 ± 1.47	0.06 ± 0.15
Gemellaceae	0.79 ± 2.88	0.94 ± 2.11	1.26 ± 1.69
Moraxellaceae	0.71 ± 3.03	0 ± 0	0 ± 0
**Genus**			
Corynebacterium	29.83 ± 33.28	3.97 ± 4.57	35.57 ± 35.68
Staphylococcus	14.62 ± 20.91	9.47 ± 20.28	8.86 ± 10.24
Prevotella	11.4 ± 22.38	20.95 ± 15.48	7.63 ± 11.95
Streptococcus	9.88 ± 14.11	25 ± 20.53	5.77 ± 6.02
Tepidiphilus	8.6 ± 17.34	5.08 ± 9.8	16.6 ± 17.7
Clostridium sensu stricto 12	5.51 ± 14.87	0.19 ± 0.44	6.04 ± 6.16
Actinobacillus	4.32 ± 16.98	0.79 ± 1.76	0.39 ± 1.03
Veillonella	3.67 ± 7.19	14.11 ± 8.48	5.08 ± 8.19
Haemophilus	3.42 ± 5.72	2.55 ± 2.9	3.61 ± 3.88
Fusobacterium	2.13 ± 4.24	4.72 ± 5.76	7.35 ± 10.27
Neisseria	2.1 ± 6.46	0.51 ± 1.13	0.23 ± 0.62
Alloprevotella	1.71 ± 4.06	11.06 ± 12.54	1.54 ± 2.49
Escherichia-Shigella	0.89 ± 5.51	0.66 ± 1.47	0.06 ± 0.15
Gemella	0.79 ± 2.88	0.94 ± 2.11	1.26 ± 1.69
Moraxella	0.71 ± 3.03	0 ± 0	0 ± 0
Aggregatibacter	0.43 ± 1.67	0 ± 0	0 ± 0

Table S6. Lefse analysis* for SARS-Cov-2 test results at different taxonomic levels.

Genus	P-values	FDR	DETECTED	NOT DETECTED	LDAscore
Thermoanaerobacterium	**0.0019528**	0.0068412	296670	872410	**-5.46**
Prevotella	**0.0022882**	0.0068412	356990	1223700	**-5.64**
Haemophilus	**0.0025655**	0.0068412	158890	654950	**-5.39**
Corynebacterium	**0.022256**	0.044512	1373100	2947600	**-5.9**
Veillonella	**0.029136**	0.046617	761810	1398000	**-5.5**
Tepidiphilus	0.14201	0.18935	1105500	0	5.74
Staphylococcus	0.84906	0.95439	2973500	1917200	5.72
Streptococcus	0.95439	0.95439	2973500	986110	6

Family	P-values	FDR	DETECTED	NOT DETECTED	LDAscore
Family III	**0.0019528**	0.0068412	296670	872410	**-5.46**
Prevotellaceae	**0.0022882**	0.0068412	356990	1223700	**-5.64**
Pasteurellaceae	**0.0025655**	0.0068412	158890	654950	**-5.39**
Corynebacteriaceae	**0.022256**	0.044512	1373100	2947600	**-5.9**
Veillonellaceae	**0.029136**	0.046617	761810	1398000	**-5.5**
Hydrogenophilaceae	0.14201	0.18935	1105500	0	5.74
Staphylococcaceae	0.84906	0.95439	2973500	1917200	5.72
Streptococcaceae	0.95439	0.95439	2973500	986110	6

Order	P-values	FDR	DETECTED	NOT DETECTED	LDAscore
**Thermoanaerobacterales**	**0.0019528**	0.0068412	296670	872410	**-5.46**
**Bacteroidales**	**0.0022882**	0.0068412	356990	1223700	**-5.64**
**Pasteurellales**	**0.0025655**	0.0068412	158890	654950	**-5.39**
**Corynebacteriales**	**0.022256**	0.044512	1373100	2947600	**-5.9**
**Veillonellales-Selenomonadales**	**0.029136**	0.046617	761810	1398000	**-5.5**
Burkholderiales	0.14201	0.18935	1105500	0	5.74
Staphylococcales	0.84906	0.95439	2973500	1917200	5.72
Lactobacillales	0.95439	0.95439	2973500	986110	6

Class	P-values	FDR	DETECTED	NOT DETECTED	LDAscore
**Thermoanaerobacteria**	**0.0019528**	0.0068646	296670	872410	-5.46
**Bacteroidia**	**0.0022882**	0.0068646	356990	1223700	-5.64
**Actinobacteria**	**0.022256**	0.043703	1373100	2947600	-5.9
**Negativicutes**	**0.029136**	0.043703	761810	1398000	-5.5
Bacilli	0.079098	0.094918	5947000	2903300	6.18
Gammaproteobacteria	0.37137	0.37137	1264400	654950	5.48

Phylum	P-values	FDR	DETECTED	NOT DETECTED	LDAscore
**Bacteroidota**	**0.0022882**	0.0091528	356990	1223700	-5.64
**Actinobacteriota**	**0.022256**	0.044512	1373100	2947600	-5.9
Firmicutes	0.13032	0.17376	7005500	5173700	5.96
Proteobacteria	0.37137	0.37137	1264400	654950	5.48

* Table shows feature ranked by their p-value, whit significant features highlighted in bold.

Table S7. Lefse analysis* for severity group at different taxonomic levels.

Genus	P-values	FDR	Detected-Ambulatory	Detected-Hospitalized	Not detected	LDAscore
Prevotella	**0.00032865**	0.0021935	121490	1770000	1223700	**5.92**
Veillonella	**0.00054839**	0.0021935	369990	3112700	1398000	**6.14**
Haemophilus	**0.0037179**	0.0099144	151750	201730	654950	**5.4**
Thermoanaerobacterium	**0.0079465**	0.015893	286860	355560	872410	**5.47**
Corynebacterium	0.071966	0.11515	1432600	1016400	2947600	5.98
Tepidiphilus	0.30076	0.40102	1171300	711110	0	5.77
Staphylococcus	0.51719	0.59108	3241200	1367500	1917200	5.97
Streptococcus	0.9983	0.9983	3224900	1465000	986110	6.05

Family	P-values	FDR	Detected-Ambulatory	Detected-Hospitalized	Not detected	LDAscore
Prevotellaceae	**0.00032865**	0.0021935	121490	1770000	1223700	**5.92**
Veillonellaceae	**0.00054839**	0.0021935	369990	3112700	1398000	**6.14**
Pasteurellaceae	**0.0037179**	0.0099144	151750	201730	654950	**5.4**
Family_III	**0.0079465**	0.015893	286860	355560	872410	**5.47**
Corynebacteriaceae	0.071966	0.11515	1432600	1016400	2947600	5.98
Hydrogenophilaceae	0.30076	0.40102	1171300	711110	0	5.77
Staphylococcaceae	0.51719	0.59108	3241200	1367500	1917200	5.97
Streptococcaceae	0.9983	0.9983	3224900	1465000	986110	6.05

Order	P-values	FDR	Detected-Ambulatory	Detected-Hospitalized	Not detected	LDAscore
**Bacteroidales**	**0.00032865**	0.0021935	121490	1770000	1223700	**5.92**
**Veillonellales-Selenomonadales**	**0.00054839**	0.0021935	369990	3112700	1398000	**6.14**
**Pasteurellales**	**0.0037179**	0.0099144	151750	201730	654950	**5.4**
**Thermoanaerobacterales**	**0.0079465**	0.015893	286860	355560	872410	**5.47**
Corynebacteriales	0.071966	0.11515	1432600	1016400	2947600	5.98
Burkholderiales	0.30076	0.40102	1171300	711110	0	5.77
Staphylococcales	0.51719	0.59108	3241200	1367500	1917200	5.97
Lactobacillales	0.9983	0.9983	3224900	1465000	986110	6.05

Class	P-values	FDR	Detected-Ambulatory	Detected-Hospitalized	Not detected	LDAscore
**Bacteroidia**	**0.00032865**	0.0016452	121490	1770000	1223700	**5.92**
**Negativicutes**	**0.00054839**	0.0016452	369990	3112700	1398000	**6.14**
**Thermoanaerobacteria**	**0.0079465**	0.015893	286860	355560	872410	**5.47**
**Bacilli**	**0.021081**	0.031621	6466100	2832500	2903300	**6.26**
Actinobacteria	0.071966	0.086359	1432600	1016400	2947600	5.98
Gammaproteobacteria	0.52785	0.52785	1323000	912840	654950	5.52

Phylum	P-values	FDR	Detected-Ambulatory	Detected-Hospitalized	Not detected	LDAscore
**Bacteroidota**	**0.00032865**	0.0013146	121490	1770000	1223700	**5.92**
Actinobacteriota	0.071966	0.14393	1432600	1016400	2947600	5.98
Firmicutes	0.27114	0.36152	7122900	6300800	5173700	5.99
Proteobacteria	0.52785	0.52785	1323000	912840	654950	5.52

* Table shows feature ranked by their p-value, whit significant features highlighted in bold.
